# Early prediction of MODS interventions in the intensive care unit using machine learning

**DOI:** 10.1186/s40537-023-00719-2

**Published:** 2023-05-04

**Authors:** Chang Liu, Zhenjie Yao, Pengfei Liu, Yanhui Tu, Hu Chen, Haibo Cheng, Lixin Xie, Kun Xiao

**Affiliations:** 1 Center of Pulmonary & Critical Care Medicine, Chinese People’s Liberation Army (PLA) General Hospital, Beijing, 100039 China; 2 School of Medicine, Nankai University, Tianjin, 300071 China; 3 Institute of Microelectronics, Chinese Academy of Sciences, Beijing, 100029 China; 4 Purple Mountain Laboratory: Networking, Communications and Security, Nanjing, 211111 China

**Keywords:** MODS, Stacked ensemble, Feature interpretation, Decision recommendation

## Abstract

**Background:**

Multiple organ dysfunction syndrome (MODS) is one of the leading causes of death in critically ill patients. MODS is the result of a dysregulated inflammatory response that can be triggered by various causes. Owing to the lack of an effective treatment for patients with MODS, early identification and intervention are the most effective strategies. Therefore, we have developed a variety of early warning models whose prediction results can be interpreted by Kernel SHapley Additive exPlanations (Kernel-SHAP) and reversed by diverse counterfactual explanations (DiCE). So we can predict the probability of MODS 12 h in advance, quantify the risk factors, and automatically recommend relevant interventions.

**Methods:**

We used various machine learning algorithms to complete the early risk assessment of MODS, and used a stacked ensemble to improve the prediction performance. The kernel-SHAP algorithm was used to quantify the positive and minus factors corresponding to the individual prediction results, and finally, the DiCE method was used to automatically recommend interventions. We completed the model training and testing based on the MIMIC-III and MIMIC-IV databases, in which the sample features in the model training included the patients’ vital signs, laboratory test results, test reports, and data related to the use of ventilators.

**Results:**

The customizable model called SuperLearner, which integrated multiple machine learning algorithms, had the highest authenticity of screening, and its Yordon index (YI), sensitivity, accuracy, and utility_score on the MIMIC-IV test set were 0.813, 0.884, 0.893, and 0.763, respectively, which were all maximum values of eleven models. The area under the curve of the deep–wide neural network (DWNN) model on the MIMIC-IV test set was 0.960, and the specificity was 0.935, which were both the maximum values of all these models. The Kernel-SHAP algorithm combined with SuperLearner was used to determine the minimum value of glasgow coma scale (GCS) in the current hour (OR = 0.609, 95% CI   0.606–0.612), maximum value of MODS score corresponding to GCS in the past 24 h (OR = 2.632, 95% CI 2.588–2.676), and maximum score of MODS corresponding to creatinine in the past 24 h (OR = 3.281, 95% CI   3.267–3.295) were generally the most influential factors.

**Conclusion:**

The MODS early warning model based on machine learning algorithms has considerable application value, and the prediction efficiency of SuperLearner is superior to those of SubSuperLearner, DWNN, and other eight common machine learning models. Considering that the attribution analysis of Kernel-SHAP is a static analysis of the prediction results, we introduce the DiCE algorithm to automatically recommend *counterfactuals* to reverse the prediction results, which will be an important step towards the practical application of automatic MODS early intervention.

**Supplementary Information:**

The online version contains supplementary material available at 10.1186/s40537-023-00719-2.

## Introduction

Multiple organ dysfunction syndrome (MODS) is defined as an acute and potentially reversible dysfunction of two or more organs induced by various factors. The incidence of MODS in adult patients admitted to ICU is 11–40%.[1, 2]. MODS is very common in critically ill patients, with a mortality rate of 44–76% [3–5]. The MODS mortality rate is related to the number of affected organs and the severity of each organ dysfunction. In cases of 2–4 organs failing, the mortality rate is 10–40%, whereas it is up to 50% in patients with cumulative five organ failure and 100% in patients with cumulative seven organ failure [6, 7].

MODS has a high mortality due to the lack of effective treatment, so early warning and intervention in the development of MODS is of great clinical importance [8]. Bose et al. determined the tags per min in the past 24 h according to IPSCC and the MODS standard proposed by Proulx et al. and added the waveform data features extracted using the spectral clustering method and used four algorithms to complete the early warning of MODS in children, in which the area under the ROC curve (AUC) of the random forest algorithm was ≥ 0.91, and the median early warning time was 22.7 h for random forest and 37 h for XGBoost models [9–12]. In addition to conventional model evaluation standards such as AUC, a new model prediction performance evaluation standard, utility scores, has been proposed, which believes that early or late warning is not helpful. [13]. Li et al. applied the utility scores to the performance comparison of sepsis early warning model for the first time; inspired by utility scores for sepsis, we proposed the utility scores of MODS [14]. In addition, the sample labels were determined based on the MODS diagnostic criterion [15, 16], and the features for model training were derived from the clinical and scoring characteristics [17–19]. Characteristics usually refer to the mathematical calculation of features, such as mean value and variance. In recent years, many studies have shown that stacked ensemble algorithms have greater predictive advantages in clinical decision support. Fan et al. used a stacked ensemble algorithm to classify normal and delayed hospitalizations in 1599 critically ill patients with spinal cord injuries [20]. Fan et al. selected three classifiers with the best performance from 91 base classifiers, and subsequently further superimposed the three classifiers into an stacked ensemble model using logistic regression classification. The AUC of the stacked ensemble model was 0.864, which was 6% higher than that of the non-ensemble learning classifier. Ko et al. developed the stacked ensemble algorithm called EDRnet based on 361 COVID-19 patients in Wuhan and applied the model to predict the death of 106 patients in three Korean medical institutions. The results demonstrated that the EDRnet provided 100% sensitivity, 91% specificity, and 92% accuracy [21]. The stacked ensemble algorithms [20, 21] achieved a high prediction performance and generalization ability because it fully utilized base classifiers, such as XGBoost and lightGBM, which were excellent for large sample sizes with multiple features, and the Bayesian neural network algorithm, which was suitable for small sample sets and effectively prevented overfitting. By integrating different classifiers, the disadvantages could be avoided, and the generality of the stacked ensemble algorithm could be considerably improved [22–24]. We have been exploring the use of a customizable neural network algorithm and non-neural network algorithms to integrate into a stacked adaptive algorithm, which has higher prediction performance. First, we need to develop a neural network algorithm with high prediction performance. Generally, the deeper the neural network is, the higher the prediction performance of the model; however, too high depths often caused the gradient disappearance or divergence of the weight of the loss function backpropagation. To solve the problem of gradient divergence, a batch normalization layer was added to the DWNN model used in this study; the batch normalization layer normalized the data before the input of each layer, which was conducive to eliminating gradient divergence and accelerating the training of the model, particularly for time-consuming stacked ensemble model training [25]. DWNN directly inputs the output of the middle layers to the “Concatenate”layer (Fig. 2), which solved the problem of the weight gradient disappearance of the far layer neural network. The loss function could propagate the direct gradient to the farthest layer, which was no longer influenced by the network depth [26]. Second, the stacked ensemble enabled the integration of multiple models with sub-optimal predictive performance into a model with optimal performance. A reasonable integration of multiple models could improve the generalization ability of the model. We use the Q-learning algorithm to determine the specific learners used by Stacked ensemble [27]. The interpretation of the stacked ensemble algorithm prediction results helped screen high-impact features and assisted doctors to complete decision-making interventions. Kernel-SHAP was a combination of the Linear LIME and Shapley value algorithms, which could be applied to all machine learning models, but Kernel-SHAP could not provide a scheme to reverse the outcome [28, 29]. Ramaravind et al. proposed that the DiCE (Diverse Counterfactual Explanations) algorithm provided various *counterfactuals* to reverse prediction result [30]. Jia et al. used the DiCE method to complete the recommendation of the reversal scheme for extubation failure in the ICU, thereby considerably reducing the risk of subjective intervention by doctors [31]. Compared with other relevant studies, our research has the following advantages. (1) Other scholars determine the stacked compensation based on experience or simply exhaustive, lacking theoretical support. However, we use the Q-learning algorithm to determine the stacked compensation algorithm. (2) There are two hypotheses in DiCE that other scholars have not tried to solve. However, we propose practical methods such as rule screening, which greatly weaken the defects of DiCE itself. (3) We propose the utility_score of MODS for the first time, which is more fair and objective for the model performance evaluation (Additional file 1: Section S1).

We mainly discussed the design scheme of model development, data processing and the idea of creating the stacked ensemble algorithm. And we also discussed Q-table for Q-learning, prediction results for models, analysis of risk factors for groups and individuals, and how to realize the integration of neural network and non-neural network models, how to use Kernel-SHAP in practical applications, and how to weaken limitations of DiCE algorithm.

## Methods

### Research program

**Fig. 1:**
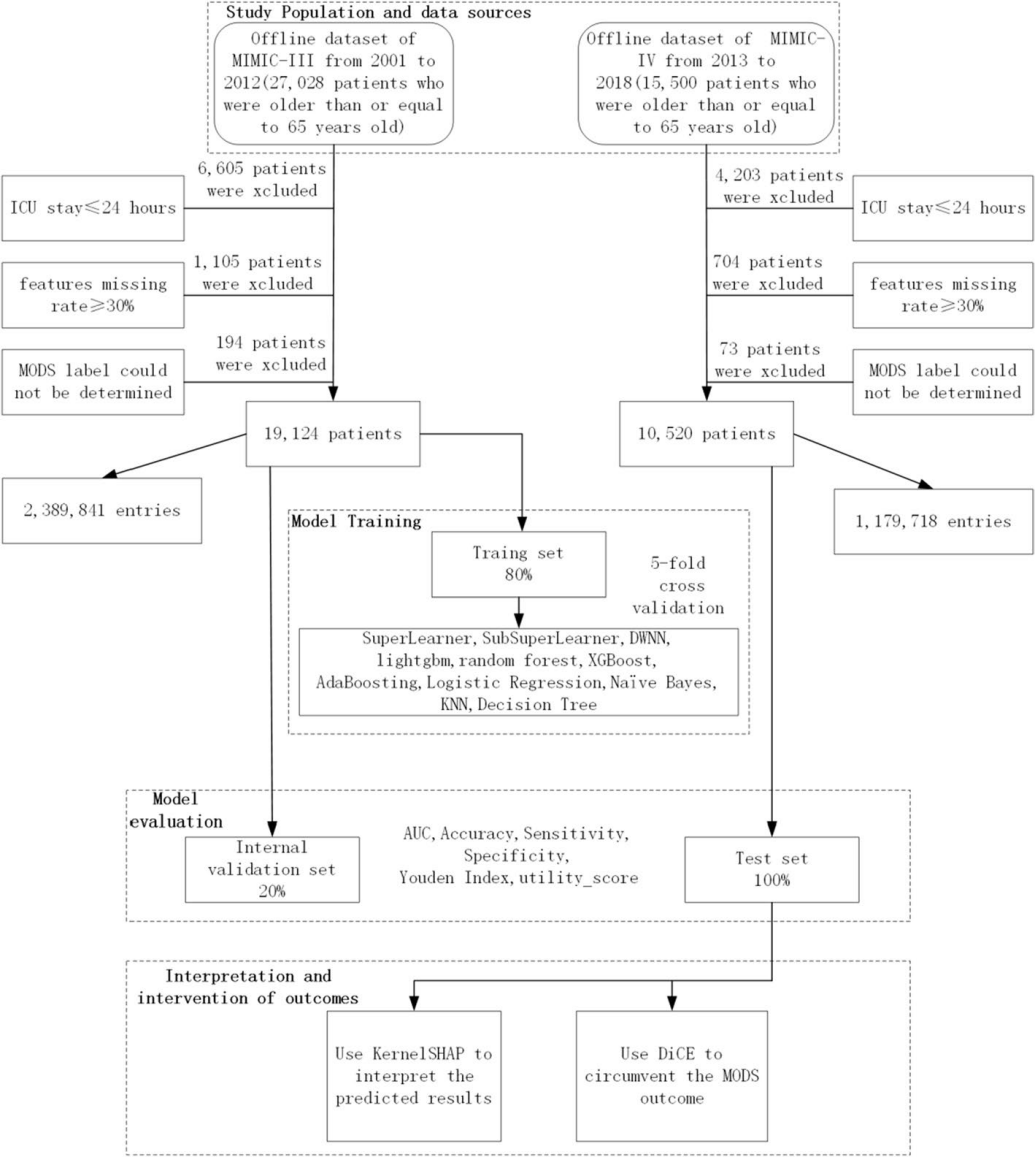
Flow Chart of research programme

As shown in Fig. 1, the study populations from 2001 to 2012 were 19,124 patients in the MIMIC-III data set, and they were ≥ 65 years old, admitted to the ICU for the first time for over 24 h, with a missing feature rate of less than 30% and had a clear outcome label; and 10,520 patients from 2013 to 2018 in the MIMIC-IV data set, who were ≥ 65 years old, admitted to the ICU for the first time for over 24 h, with a missing feature rate of less than 30% and had a clear outcome label. An entry is an sample, whose candidate features comes from a patient in an hourly time window, with 2,389,841 entries for 19,124 patients in MIMIC-III and 1,179,718 entries for 10,520 patients in MIMIC-IV. While the label of the entry is whether MODS occurred in the current hourly window, increasing in 12-h increments. When the label is occurrence of MODS, it was a positive entry, otherwise, it was a negative entry. We randomly considered 80% of the entries corresponding to the number of patients in MIMIC-III as the training set, and 20% as the internal validation data set. Entries corresponding to 10,520 patients in MIMIC-IV were used as the test set. After completing the five-fold cross-validation training of 11 models such as SuperLearner, the evaluation of the models was completed on the internal validation set and the test set, and the evaluation indicators included AUC and accuracy. Finally, Kernel-SHAP and DiCE were used to complete the interpretation and intervention of the prediction results of the test set.

### Feature selection and data processing

The candidate features were derived from the clinical features and scoring characteristics. For the cohort data of each patient, the forward or backward interpolation method is used to complete the interpolation of clinical features such as total bilirubin and creatinine. The scoring characteristics of the organs of MODS are calculated according to clinical features, so there is no interpolation for the scoring characteristics (Additional file 1: Section S2). To accelerate the convergence of the model training, it was required to standardize the entries and use the Gaussian distribution normalization method for obtaining the mean value, μ, and normal deviation, σ, of the MIMIC-III training set and subsequently apply the obtained μ and σ to the normalization of the MIMIC-III and MIMIC-IV test sets.

### Machine learning

**Fig. 2:**
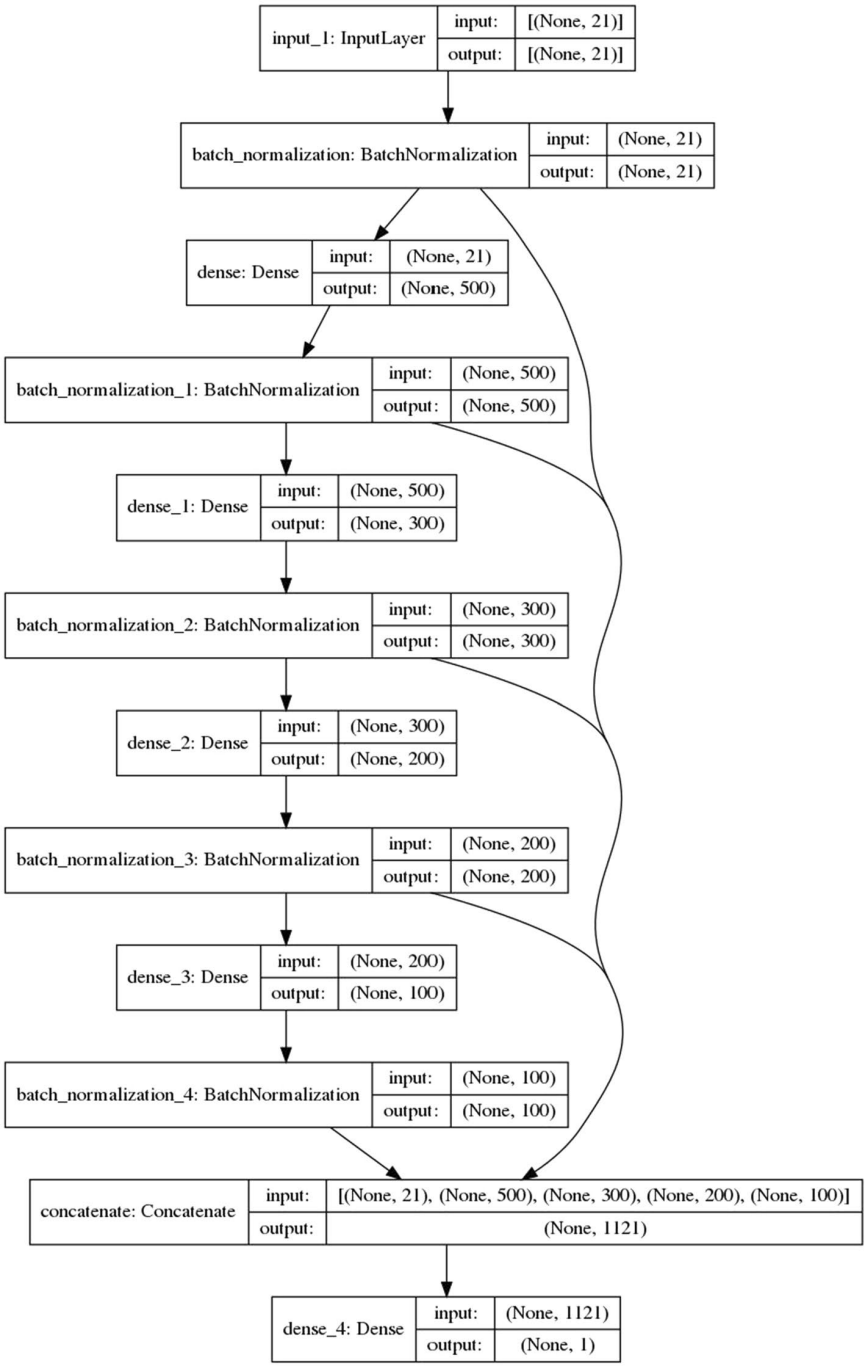
Structure of DWNN

**Fig. 3:**
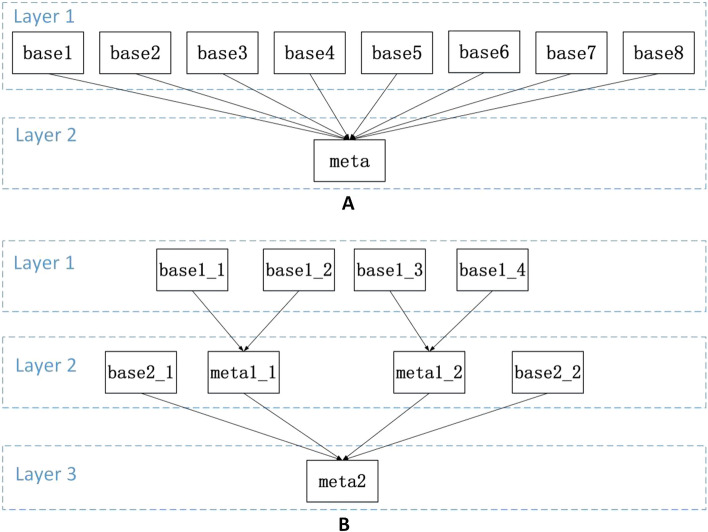
Frameworks of stacked ensembles

The deep neural network algorithm generally has a higher prediction performance than the single non-neural network algorithm, so we designed the MODS early warning algorithm, DWNN, strictly according to the requirements of neural network modeling (Fig. 2). In addition, we developed eight conventional machine learning algorithms based on the same MIMIC-III training set: KNN, lightgbm, Decision Tree, Naïve Bayes, random forest, XGBoost, AdaBoosting, and Logistic Regression. These algorithms had five-fold cross-validation and parameter optimization. The results of the MIMIC-III test set showed that DWNN model is one of the top three models with the best performance (Table 4). This study used the keras encapsulating interface of sklearn to encapsulate DWNN into the classifier interface of sklearn, and then used the interface module from sklearn to integrate multiple models. The stacked ensemble model could directly invoke and complete the general and individual sample interpretation using the Kernel-SHAP algorithm interface. The stacked ensemble enabled the integration of multiple models with sub-optimal predictive performance into a model with optimal performance. Conventional non-neural network machine learning algorithms included eight types: logistic regression, random forest, Bayesian, XGBoost, lightGBM, etc. A limitation of stacked ensemble algorithm is the difficulty of optimizing the integration framework. Two stacked ensemble schemes were used in this study (Fig. 3).

Figure 3A is a two-layer stacked ensemble structure called SuperLearner composed of base learners and meta-learners, where base_1 ~ base_8 are the base learners, and the predictive probabilities of the base learners are used as the input features of the meta-learner. Figure 3B is a customizable three-layer stacked ensemble structure called SubSuperLearner. The predictive probabilities of base1_1 and base1_2 are used as the input features of meta1_1. The predictive probabilities of base1_3 and base1_4 are used as the input features of meta1_2. The predictive probabilities of meta1_1, meta1_2, base2_1 and base2_2 are used as the input features of meta2. For Fig. 3A, B, each learner can be selected from nine algorithms (excluding SuperLearner and SubSuperLearner). If you use the exhaustive method to determine which algorithm the learner is to achieve maximum AUC for SuperLearner or SubSuperLearner, you must exhaustive 9 ^ 9 = 387420489 times. The adoption of exhaustive selection is time-intensive and was not in line with reality. Herein, we used the Q-learning algorithm with ε-greedy strategy to determine each base learner and meta learner [32]. We have completed the pseudo-code of the Q-learning algorithm and its detailed description (Additional file 1: Sect. S3).

### Statistical analysis

SPSS 20.0 software was used for statistical analysis, which was in line with the normal distribution characteristics and was expressed as ± standard deviation of the mean value ($\overline{x} \pm s$), and the inter-group comparison was performed using the $t$ test; comparisons of the counting data groups were examined using the X^2^ test. The dependent variable was whether patients had MODS or not, and the independent variable was the index screened by the Univariate Analysis of the influencing factors of MODS. The related index was screened using multivariate logistic regression analysis, and the difference was statistically significant when P < 0.001.

We used Python 3.6 analysis and loaded third-party modules, such as sklearn, XGBoost, torch, shap, and imblearn. The AUC, accuracy, sensitivity, specificity, YI, and the utility_score of SuperLearner, SubSuperLearner, and DWNN models on the internal validation set and test set were calculated. To eliminate the random error of a single trial, this study was repeated 10 times. For the definition of utility_score, please refer to Additional file 1: Section S1.

## Results

### Univariate factor logistic regression analysis between groups

The scores for the six organs of MODS can be addressed by clinical features (Additional file 1: Section S2). Candidate features include the mean, maximum and minimum for all clinical features such as total bilirubin and creatinine, as well as the scoring characteristics for the six organs of MODS within an hourly window. The total number of candidate features listed in the latest manuscript was 37. After one-way logistic regression analysis, 21 features were selected with statistical significance (P < 0.001) (Table 1). The factors with larger contribution values were currentMinGcs, gcs24HoursMods, and renal24HoursMods (Table 2).

**Table 1:** Comparison of characteristics between MODS and non-MODS groups

Features	MODS-yes (n = 1,960,021)	MODS-no (n = 429,820)	χ^2^/t	*P*
CurrentHour	172.26 ± 236.283	104.81 ± 157.329	− 182.856	< 0.001
CurrentMaxHr	85.248 ± 17.221	80.748 ± 14.839	162.591	< 0.001
CurrentMaxDopamine	5.573 ± 5.576	5.679 ± 5.792	11.491	< 0.001
CurrentMinOi	252.419 ± 108.505	249.183 ± 111.431	− 18.033	< 0.001
CurrentMinGcs	10.899 ± 3.5792	14.686 ± 1.1529	701.963	< 0.001
CurrentMaxLactate	1.744 ± 1.404	1.723 ± 1.4259	− 9.004	< 0.001
CurrentMaxCreatinine	140.735 ± 118.691	90.040 ± 54.246	− 280.231	< 0.001
CurrentMaxBilirubin	247.066 ± 120.868	148.548 ± 332.848	− 80.377	< 0.001
CurrentMinPlatelet	224.990 ± 130.619	247.066 ± 120.868	104.052	< 0.001
CurrentCardiovascularMods	0.105 ± 0.507	0.009 ± 0.122	− 125.529	< 0.001
CurrentRespiratoryMods	1.373 ± 1.122	1.330 ± 1.090	23.750	< 0.001
CurrentRenalMods	0.777 ± 0.974	0.288 ± 0.544	− 326.096	< 0.001
CurrentGcsMods	1.784 ± 1.343	0.203 ± 0.537	− 776.422	< 0.001
CurrentHepaticMods	1.792 ± 1.125	1.628 ± 1.002	90.782	< 0.001
CurrentHematologicMods	0.317 ± 0.717	0.082 ± 0.336	− 214.788	< 0.001
Cardiovascular24HoursMods	0.103 ± 0.487	0.007 ± 0.112	− 208.112	< 0.001
Respiratory24HoursMods	1.361 ± 1.105	1.325 ± 1.011	23.620	< 0.001
Renal24HoursMods	0.766 ± 0.954	0.276 ± 0.524	− 320.051	< 0.001
gcs24HoursMods	1.773 ± 1.329	0.201 ± 0.529	− 766.399	< 0.001
Hepatic24HoursMods	1.790 ± 1.113	1.615 ± 0.991	89.770	< 0.001
Hematologic24HoursMods	0.313 ± 0.713	0.0792 ± 0.333	− 209.780	< 0.001

CurrentHour (h) indicates the cumulative duration of ICU admission; currentMaxHr (time/min) indicates the maximum heart rate in the current hour; currentMaxDopamine (UG/kg/min) indicates the maximum value of dopamine in the current hour; CurrentMinOi indicates the minimum oxygenation index in the current hour; currentMinGcs (1) indicated the minimum Glasgow index in the current hour; currentMaxLactate (mmol/L) indicates the maximum serum lactic acid in the current hour; CurrentMaxCreatinine (umol/L) indicates the maximum value of creatinine in the current hour; currentMaxBilirubin (umol/L) indicated the maximum value of total bilirubin in the current hour; CurrentMinPlatelet (109) indicates the minimum value of platelets in the current hour; CurrentCardiovas cularMods indicates the maximum value of the MODS score corresponding to the cardiovascular system in the current hour, that is, max (the MODS score corresponding to currentMaxDopamine, the MODS score corresponding to currentMaxLactate, the MODS score corresponding to currentMaxHr); currentRespiratoryMods indicates the MODS score corresponding to currentMinOi in the current hour; CurrentRenalMods indicates the MODS score corresponding to currentMaxCreatinine in the current hour; currentGcsMods indicates the MODS score corresponding to currentMinGcs in the current hour; currentHepaticMods indicates the MODS score corresponding to currentMaxBilirubin in the current hour; CurrentHematologicMods indicates the MODS score corresponding to currentMinPlatelet in the current hour; cardiovascular24HoursMods indicates the maximum value of currentCardiovas cularMods in the past 24 h; respiratory24HoursMods indicates the maximum value of currentRespiratoryMods in the past 24 h; renal24HoursMods indicates the maximum value of currentRenalMods in the past 24 h; gcs24HoursMods indicates the maximum value of currentGcsMods in the past 24 h; hepatic24HoursMods indicates the maximum value of currentHepaticMods in the past 24 h; hematologic 24HoursMods indicates the maximum value of currentHematologicMods in the past 24 h

**Table 2:** Logistic regression analysis of factors affecting MODS occurrence

Features	*β*	*S.E*	*Wald*	*P*	*OR*	*95%CI*
currentHour	0.000	0.000	299.352	< 0.001	1.000	0.995–1.005
currentMaxHr	0.012	0.000	5256.416	< 0.001	1.009	1.006–1.012
currentMaxDopamine	− 0.002	0.000	19.395	< 0.001	1.308	1.305–1.311
currentMinOi	0.000	0.000	52.850	< 0.001	0.881	0.878–0.884
currentMinGcs	− 0.197	0.006	1178.851	< 0.001	0.609	0.606–0.612
currentMaxLactate	− 0.018	0.002	93.723	< 0.001	1.208	1.204–1.212
currentMaxCreatinine	0.002	0.000	395.423	< 0.001	1.140	1.135–1.145
currentMaxBilirubin	0.000	0.000	1.353	0.245	1.000	0.996–1.004
currentMinPlatelet	0.000	0.000	0.788	0.375	1.001	0.994–1.008
currentCardiovascularMods	0.638	0.016	1589.776	< 0.001	1.892	1.834–1.953
currentRespiratoryMods	− 0.060	0.005	166.921	< 0.001	1.160	1.163–1.157
currentRenalMods	0.243	0.010	577.973	< 0.001	1.275	1.250–1.300
currentGcsMods	0.826	0.012	4984.566	< 0.001	1.284	1.233–1.337
currentHepaticMods	− 0.024	0.003	67.270	< 0.001	1.180	1.177–1.183
currentHematologicMods	0.334	0.010	1231.870	< 0.001	1.396	1.371–1.423
cardiovascular24HoursMods	0.801	0.005	21322.022	< 0.001	1.728	1.705–1.752
respiratory24HoursMods	0.668	0.003	37779.847	< 0.001	1.851	1.838–1.864
renal24HoursMods	1.290	0.006	42944.155	< 0.001	2.632	2.588–2.676
gcs24HoursMods	0.825	0.003	69569.494	< 0.001	3.281	3.267–3.295
hepatic24HoursMods	1.008	0.004	59456.583	< 0.001	1.941	1.919–1.963
hematologic24HoursMods	1.179	0.011	12531.788	< 0.001	1.550	1.583–1.517

### Q-table and ROC curves

**Fig. 4:**
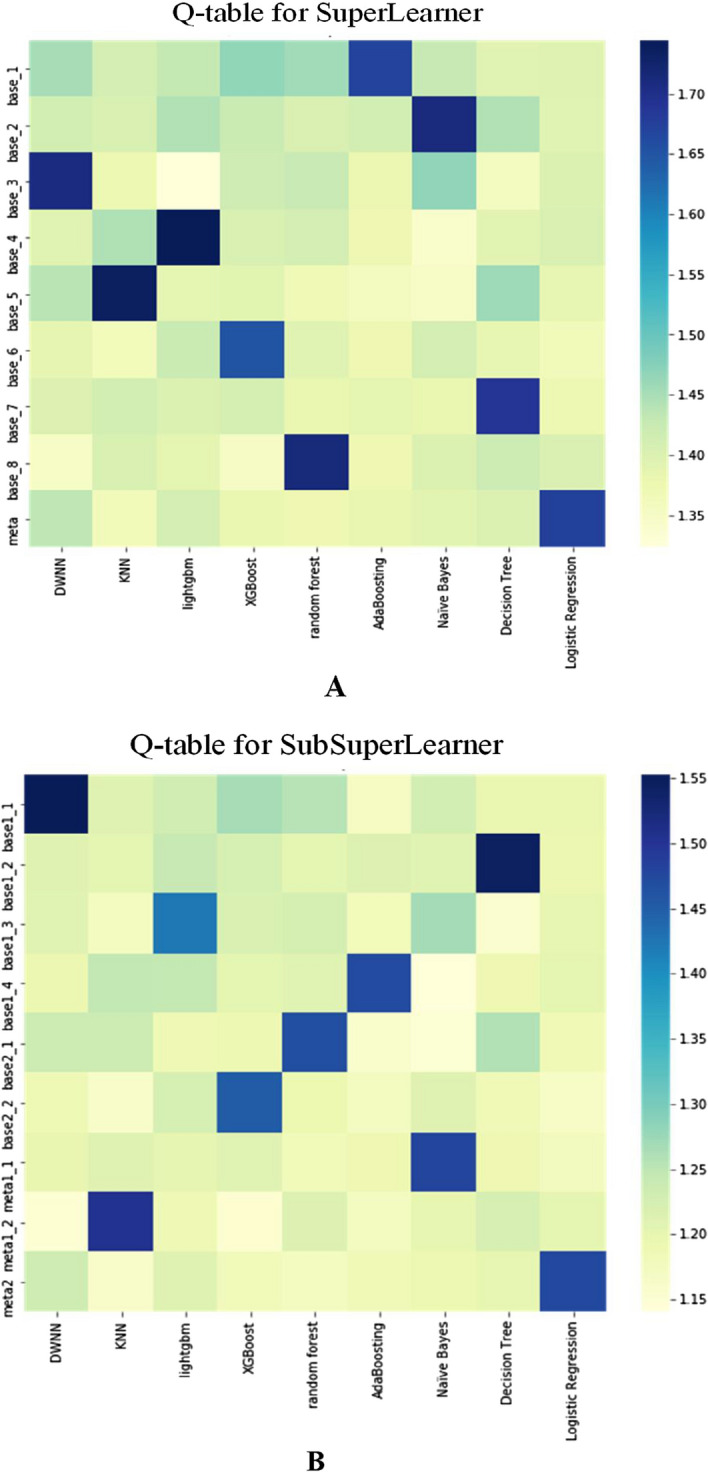
Q-table of SuperLearner and SubSuperLearner

From the 2,389,841 samples of MIMIC-III, 50,0000 samples were randomly selected for Q-learning training, and the StackingClassifier of the third-party module mlxtend was used to build the stacked ensemble model. The value of each parameter was $\gamma = 0.85,$ $\varepsilon = 0.9,$ and $\alpha = 0.1$, and the Q-learning training was terminated after 5000 iterations, and the Q-tables of SuperLearner and SubSuperLearner were obtained (Fig. 4).

The trained Q-learning algorithm would store the information selected by the learner that obtained the maximum reward in the Q-table, and we were only required to determine the learner corresponding to the maximum value of each row in the Q-table for determining which learner was selected in the rectangular box, as shown in Fig. 3A, B. As shown in Fig. 4, the color of AdaBoosting was the darkest in the state base_1 row, so the agent should select the AdaBoosting base learner at the base_1 position. Thus, the SuperLearner structure should be base_1 selecting AdaBoosting, base_2 selecting Na Naïve Bayes, base_3 selecting DWNN, base_4 selecting lightgbm, base_5 selecting KNN, base_6 selecting XGBoost, base_7 selecting decision tree, base_7 selecting random, and meta selecting logistic regression. As shown in Fig. 4B, the SubSuperLearner structure is base1_1 selecting DWNN, base1_2 selecting Decision Tree, base 1_3 selecting lightgbm, base1_4 selecting AdaBoosting, base2_1 selecting random forest, base2_2 selecting XGBoost, meta1_1 selecting Na Naïve Bayes, meta1_2 selecting KNN, and meta2 selecting logistic regression.

**Fig. 5:**
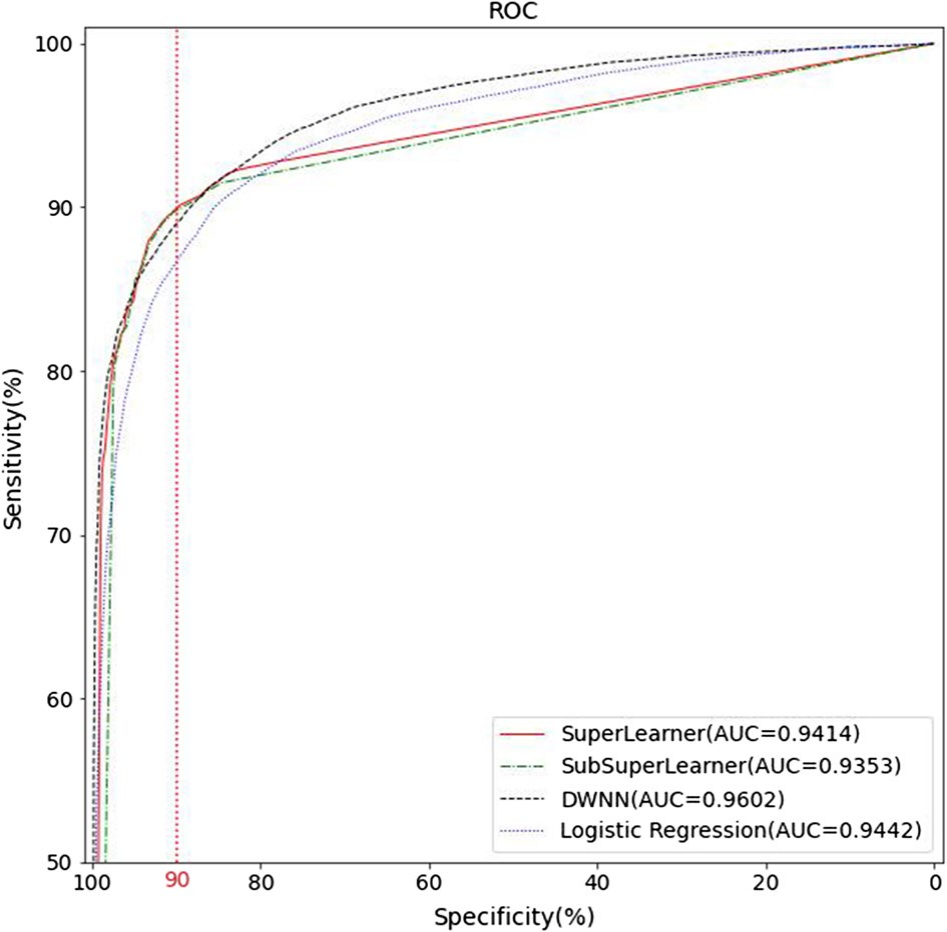
Fig. 5 ROC curves

After SuperLearner and SubSuperLearner were determined, 10 independent trials were completed, and the ROC curves for them obtained in the MIMIC-IV test set is shown in Fig. 5. The sensitivity values corresponding to different specificities in Fig. 5 are listed in Table 3.

**Table 3:** Sensitivity values corresponding to different specificities

Model	Specificities with different Sensitivities					AUC
	0.95	0.90	0.85	0.80	0.75	
SuperLearner	0.8746	0.9043	0.9224	0.9268	0.9315	0.9413 ± 0.014
SubSuperLearner	0.8701	0.9042	0.9151	0.9200	0.9251	0.9352 ± 0.012
DWNN	0.8520	0.8924	0.9160	0.9357	0.9510	0.9603 ± 0.013
Logistic Regression	0.8239	0.8735	0.9097	0.9294	0.9406	0.9441 ± 0.013

As shown in Table 4, DWNN had the best performance in nine algorithms (excluding SuperLearner and SubSuperLearner). Figure 5 shows the ROC curves of SuperLearner, SubSuperLearner, DWNN, and logistic regression, and the maximum AUC of DWNN was 0.9602. Table 3 shows that the SuperLearner sensitivity achieved the maximum value when the specificity was ≥ 85%, and the DWNN sensitivity achieved the maximum value when the specificity was ≤ 80%.

**Table 4:** Comparison of the prediction ability of each model ()

	MIMIC-III						MIMIC-IV					
Models	AUC	Accuracy	Sensitivity	Specificity	YI	Utility_score	AUC	Accuracy	Sensitivity	Specificity	YI	utility_score
Super learner	0.949 ± 0.014	0.902 ± 0.013	0.896 ± 0.014	0.930 ± 0.012	0.826 ± 0.011	0.780 ± 0.011	0.941 ± 0.014	0.893 ± 0.011	0.884 ± 0.011	0.929 ± 0.013	0.813 ± 0.014	0.763 ± 0.011
SubSuperLearner	0.942 ± 0.012	0.901 ± 0.014	0.896 ± 0.012	0.928 ± 0.014	0.824 ± 0.014	0.778 ± 0.012	0.935 ± 0.012	0.888 ± 0.012	0.880 ± 0.014	0.925 ± 0.014	0.809 ± 0.012	0.760 ± 0.011
DWNN	0.967 ± 0.012	0.891 ± 0.011	0.881 ± 0.011	0.939 ± 0.013	0.820 ± 0.012	0.705 ± 0.015	0.960 ± 0.013	0.882 ± 0.014	0.869 ± 0.012	0.935 ± 0.012	0.804 ± 0.011	0.690 ± 0.015
lightgbm	0.964 ± 0.015	0.887 ± 0.013	0.879 ± 0.012	0.927 ± 0.012	0.806 ± 0.012	0.759 ± 0.013	0.959 ± 0.014	0.884 ± 0.014	0.873 ± 0.012	0.930± 0.015	0.903 ± 0.012	0.738 ± 0.015
random forest	0.963 ± 0.014	0.886 ± 0.012	0.876 ± 0.012	0.932 ± 0.011	0.808 ± 0.015	0.734 ± 0.012	0.958 ± 0.012	0.878 ± 0.014	0.863 ± 0.015	0.943 ± 0.012	0.806 ± 0.012	0.711 ± 0.012
XGBoost	0.959 ± 0.014	0.887 ± 0.012	0.882 ± 0.012	0.910 ± 0.012	0.792 ± 0.013	0.756 ± 0.014	0.953 ± 0.012	0.878 ± 0.014	0.868 ± 0.013	0.921 ± 0.011	0.789 ± 0.012	0.730 ± 0.012
AdaBoosting	0.958 ± 0.014	0.873 ± 0.011	0.868 ± 0.012	0.897 ± 0.012	0.765 ± 0.012	0.726 ± 0.011	0.954 ± 0.013	0.867 ± 0.013	0.853 ± 0.012	0.928 ± 0.013	0.781 ± 0.012	0.705 ± 0.013
Logistic Regression	0.955 ± 0.014	0.883 ± 0.014	0.875 ± 0.012	0.919 ± 0.015	0.794 ± 0.015	0.678 ± 0.013	0.944 ± 0.013	0.872 ± 0.014	0.862 ± 0.015	0.919 ± 0.013	0.781 ± 0.012	0.657 ± 0.015
Naïve Bayes	0.938 ± 0.012	0.834 ± 0.013	0.815 ± 0.015	0.923 ± 0.011	0.738 ± 0.012	0.657 ± 0.014	0.936 ± 0.013	0.810 ± 0.014	0.779 ± 0.012	0.942 ± 0.012	0.721 ± 0.015	0.620 ± 0.012
KNN	0.938 ± 0.013	0.850 ± 0.013	0.833 ± 0.014	0.932 ± 0.012	0.765 ± 0.012	0.616 ± 0.011	0.920 ± 0.011	0.828 ± 0.012	0.804 ± 0.012	0.932 ± 0.015	0.736 ± 0.014	0.608 ± 0.013
Decision Tree	0.928 ± 0.012	0.852 ± 0.014	0.839 ± 0.011	0.912 ± 0.012	0.751 ± 0.015	0.667 ± 0.014	0.914 ± 0.011	0.823 ± 0.012	0.798 ± 0.015	0.926 ± 0.014	0.724 ± 0.011	0.610 ± 0.012

### Model evaluation

The AUC, accuracy, sensitivity, specificity,YI, and utility_score of various machine learning models are listed in Table 4. As shown in Table 4, SuperLearner obtained the highest screening authenticity in Accuracy, Sensitivity, YI and utility_score for the SuperLearner model; DWNN achieved maximum values of AUC and specificity.

### Explanation and intervention of SuperLearner

**Fig. 6:**
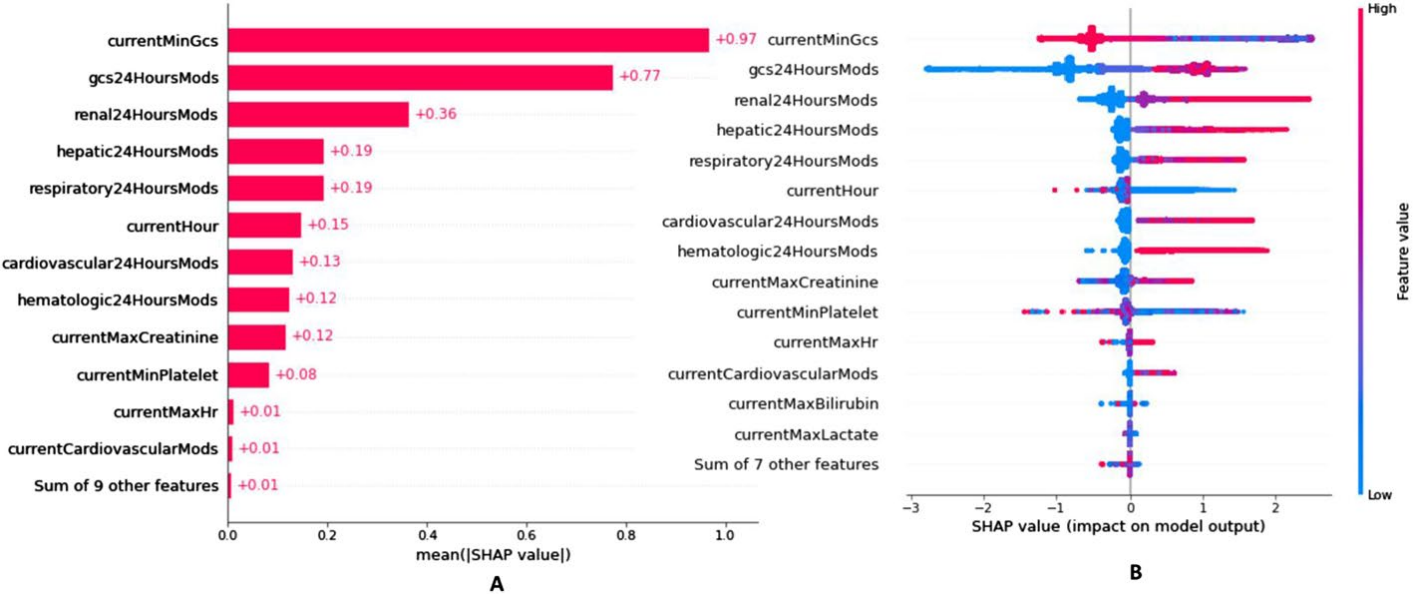
Contribution of group factors

**Fig. 7:**
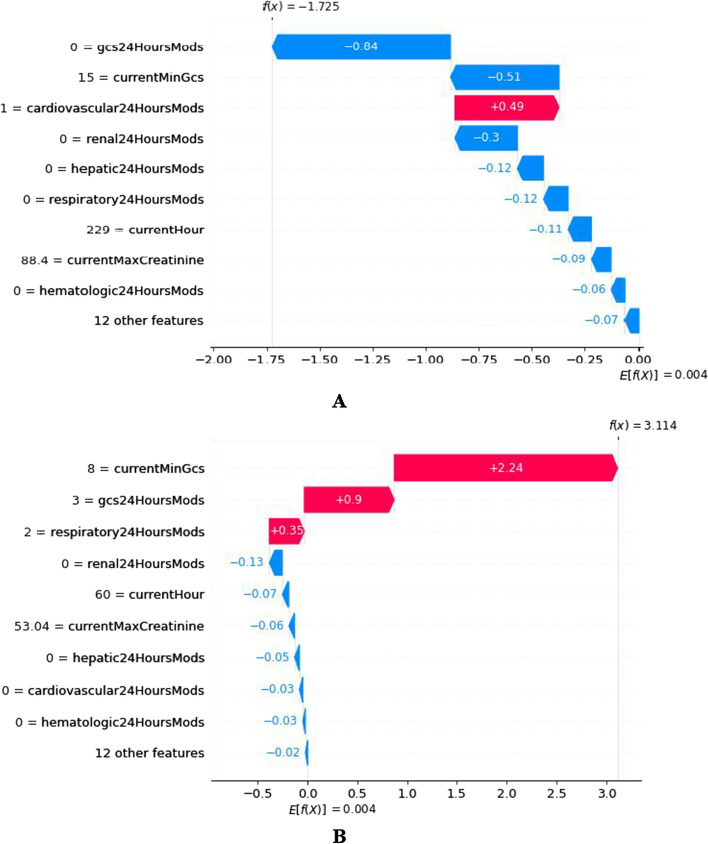
Contribution of individual factors

SuperLearner is a stacked ensemble model that contains eight non-neural network algorithms and one deep neural network algorithm. The Kernel-SHAP algorithm could quantify the contribution of the general (population) factors (Fig. 6) and local (individual) factors of SuperLearner (Fig. 7).

Figure 6A shows that currentMinGcs, gcs24HoursMods, and renal24HoursMods have the highest population contribution values, which is consistent with Table 2. The blue in Fig. 6B indicates that the observed value of the feature factor is small, and the red indicates that the observed value of the feature factor is large. The abscissa is the SHAP value. Generally, the larger the SHAP value, the greater the MODS risk. Figure 6B shows that currentMinGcs is negatively correlated with MODS, and the corresponding OR in Table 3 is also less than 1. The factors for calculating MODS scores are positively correlated with MODS occurrence, and the corresponding OR values in Table 2 are also greater than 1.

Causality cannot be derived directly from the statistically determined risk factors. Therefore, we study the correlation between risk factors and the predicted outcomes. We can regard contributions as correlations. An entry is a sample. We use a simple sampling method to randomly select a sample with a predictied outcomes of MODS and a sample with a predicted outcome of no-MODS from test set. For Fig. 7, the abscissa represents the risk factor contribution value (SHAP value), and the ordinate represents the risk factor (feature) with the observation value. If the risk factor contribution value is positive, it indicates that the factor is positively correlated with the prediction result, and the color is red; otherwise, the factor is negative correlated with the prediction result, and the color is blue. It should be noted that all samples in the test set are involved in SHAP analysis, and then the risk factor contribution value of each sample is obtained. *f*(*x*) in Fig. 7 contains the sum of SHAP values of all risk factors in the current sample (Additional file 1: Section S4); “*E*[*f*(*x*)] = 0.004” means the mean value of a of all samples including train set and the above test samples is 0.004 in Fig. 7. As shown in Fig. 7A, only currentCardiovascularMods is an unfavorable factor, and the rest are favorable factors. It was the 229th hour of ICU admission (currentHour = 229) for patient A, who had a mild cardiovascular disease (currentCardiovascularMods = 1). However, the consciousness was particularly clear, and the conversational and motor abilities were normal (currentMinGcs = 0 and gcs24HoursMods = 0) on the final day. So the patient A did not develop MODS at 241 h, which was consistent with the patient’s symptoms. Figure 7B shows that it was the 60th hour of ICU admission (currentHour = 60) for patient B. Patient B had three adverse factors: in the past 24, patient B was unconscious and had severe impairments of movement and respiratory system (gcs24HoursMods = 8, currentMinGcs = 3, and respiratory24HoursMods = 2). So the patient B developed MODS at 72 h, which was consistent with the patient’s symptoms.

For patient B who required immediate intervention, we used the DiCE algorithm to automatically recommend *counterfactuals* for the doctors to select, one of which is shown in Table 5.

**Table 5:** Example of generated counterfactual for the specific patient in Fig. 7B

Features	Original input	Counterfactual example
currentMinGcs	8	15
gcs24HoursMods	3	2
respiratory24HoursMods	2	1
renal24HoursMods	0	–
currentHour	60	–
currentMaxCreatinine	53.04	–
hepatic24HoursMods	0	–
cardiovascular24HoursMods	0	–
hematologic24HoursMods	0	–
12 other features	–	–
Predicted outcome	0.97	0.11

The dashes in Table 5 indicate that the factors remain unchanged. We could sample verbal arousal, physical stimulation, and medication to increase the patient's current mental clarity, speech, and motor ability, changing the currentMinGcs value to 15 and the gcs24HoursMods value from 3 to 2. In addition, the respiratory24HoursMods was changed from 2 to 1 with ventilator use. After modification of the observed values of the above factors, the probability of MODS in patients was reduced from 0.97 to 0.11. In principle, patients would avoid MODS occurrence at the 72nd hour. Only one scheme was provided in Table 5. DiCE recommended multiple schemes, and doctors would select the most cost-effective scheme according to the actual situation of patients.

## Discussion

In this study, we developed the SuperLearner algorithm that combined the non-neural network algorithms and deep learning algorithm. We first use the "kerasClassifier" interface of tensorflow to package the customized DWNN model into a machine learning model available to sklearn. Then we use the "StackingClassifier" module of the third-party library "mlxtend" to build the candidate stacked ensemble model. Then the stacked ensemble enabled the integration of multiple models with sub-optimal predictive performance into a model with optimal performance. This study uses Q-learning to determine the SuperLearner and SubSuperLearner. Here we use the AUC of the candidate model as reward. Of course, we can also use YI or utility_score as reward. This study proposes for the first time to use the utility_score of MODS to evaluate the prediction performance for MODS early warning models. This research eliminates the imbalance of sample categories by setting category weights. In fact, the EasyEnsemble method can also be used to make full use of data to improve the classification ability of the model and reduce the bias of the model [33, 34]. In addition to Q-learning algorithm, genetic algorithm is also excellent in determining stacked ensemble algorithm.

There are supervised and unsupervised analyses when using Kernel-SHAP to analyze the attribution of the prediction results. The user first uses the training set and the trained early warning model to train the Kernel-SHAP model and this process is the supervised analysis for Kernel-SHAP. When conducting production application, our samples are not labeled and this process is unsupervised analysis for Kernel-SHAP. We need use not only the trained model to predict the results, but also the trained Kernel-SHAP model to determine the individual risk factor contribution values corresponding to the prediction results. Of course, all production applications of the Shapley Value algorithm require the above steps. "*E*[*f*(*x*)] = 0.004" in Fig. 7 shows that the unlabeled samples from the production environment will train Kernel-SHAP again. If the same sample is tested many times by Kernel-SHAP, this will inevitably lead to model deviation in Kernel-SHAP. Considering the complexity of the algorithm improvement, we directly use the backup trained Kernel-SHAP, and then cover the current Kernel-SHAP after predicting the individual sample of the production environment. In addition, if we can establish the relationship between the value of *f*(*x*) such as in Fig. 7 and probability prediction results, we can only use Kernel-SHAP to complete production applications without deploying trained early warning models. So far, we can draw the conclusion that the larger *f*(*x*) is, the more likely MODS will occur. This is very interesting and will be the focus of the next research step.

DiCE provides *counterfactuals* for reversing predicted outcomes on the premise of considering plausibility and diversity (Additional file 1: Section S5). The use of DiCE requires two assumptions. First, DiCE assumes that there is no dependence between features. Second, DiCE assumes that the prediction results can be reversed as long as *counterfactuals* are implemented within the early warning period, ignoring the time dimension. For hypothesis 1, multiple rules can be set, such as currentMinGcs <  = 6, currentGcsMods = 4, etc. to conduct a second round of screening for *counterfactuals*. For hypothesis 2, it is difficult to implement the current algorithm. We can only suggest that patients to implement *counterfactuals* as soon as possible to increase the possibility of reversing the predicted outcome.

## Conclusion

In this study, the non-neural network algorithm and customizable neural network algorithm were integrated into a two-layer stacked ensemble structure called SuperLearner and a three-layer stacked ensemble structure called SubSuperLearner. Compared to the base learners, we found that the screening ability of the two stacked ensemble structures exceed any one of them. In terms of model performance evaluation, we added utility_score of MODS for the first time in all MODS-related studies. In order to determine base learners in the two stacked ensemble structures, we innovatively used Q-learning to determinate them. In addition, we applied Kernel-SHAP to complete the attribution analysis of the prediction results of the stacked ensemble model, and give tips on the use of Kernel-SHAP for production applications. Considering that the attribution analysis of Kernel-SHAP is a static analysis of the prediction results, we introduced the DiCE algorithm to automatically recommend *counterfactuals* to reverse the prediction results, which will be an important step towards the practical application of fully automatic MODS early intervention.

## Supplementary Information

**Additional file 1: Figure S1.** Diagrams of utility of positive and negative predictions for MODS and non-MODS. **Table S1.** The modified multiple organ dysfunction syndrome (MODS) score. **Table S2.** The Q-table for SuperLearner. **Table S3.** The Q-table for SubSuperLearner.

## Data Availability

The datasets generated and analyzed during the current study are not publicly available for privacy reasons but anonymized data are available from the corresponding author on reasonable request.
