# Supplementary material for: Early prediction of MODS interventions in the intensive care unit using machine learning
Source: J Big Data. 2023 May 4;10(1):55. doi: 10.1186/s40537-023-00719-2 (PMC10158675; doi:10.1186/s40537-023-00719-2)
Supplement: Supplementary file 1 — Additional file 1: Figure S1. Diagrams of utility of positive and negative predictions for MODS and non-MODS. Table S1. The modified multiple organ dysfunction syndrome (MODS) score. Table S2. The Q-table for SuperLearner. Table S3. The Q-table for SubSuperLearner. [file 40537_2023_719_MOESM1_ESM.docx]

Additional file

1. **Utility_score for MODS**

We scored each algorithm’s predictions using a novel evaluation metric that we created for the MODS. Each algorithm made a binary MODS prediction for each hourly time window in each patient during the entire period of first admission to ICU. To evaluate each algorithm, we first defined a score for each prediction and then aggregated these scores over all hourly time windows and all patient records. Given an algorithm’s prediction for an hourly time window t in a patient record s, we define a score

$$U(s,t)=\left\{\begin{matrix} U_{\text{TP}}(s,t), \text{positive} \text{prediction} \text{at} \text{time} t \text{for} \text{a} \text{patient} s \text{with} \text{MODS}, \\ U_{\text{FP}}(s,t), \text{positive} \text{prediction} \text{at} \text{time} t \text{for} \text{a} \text{patient} s \text{with} \text{non}-\text{MODS}, \\ U_{\text{FN}}(s,t), \text{negative} \text{prediction} \text{at} \text{time} t \text{for} \text{a} \text{patient} s \text{with} \text{MODS}, \\ U_{\text{TN}}(s,t), \text{negative} \text{prediction} \text{at} \text{time} t \text{for} \text{a} \text{patient} s \text{with} \text{non}-\text{MODS}, \end{matrix}\right. (1)$$

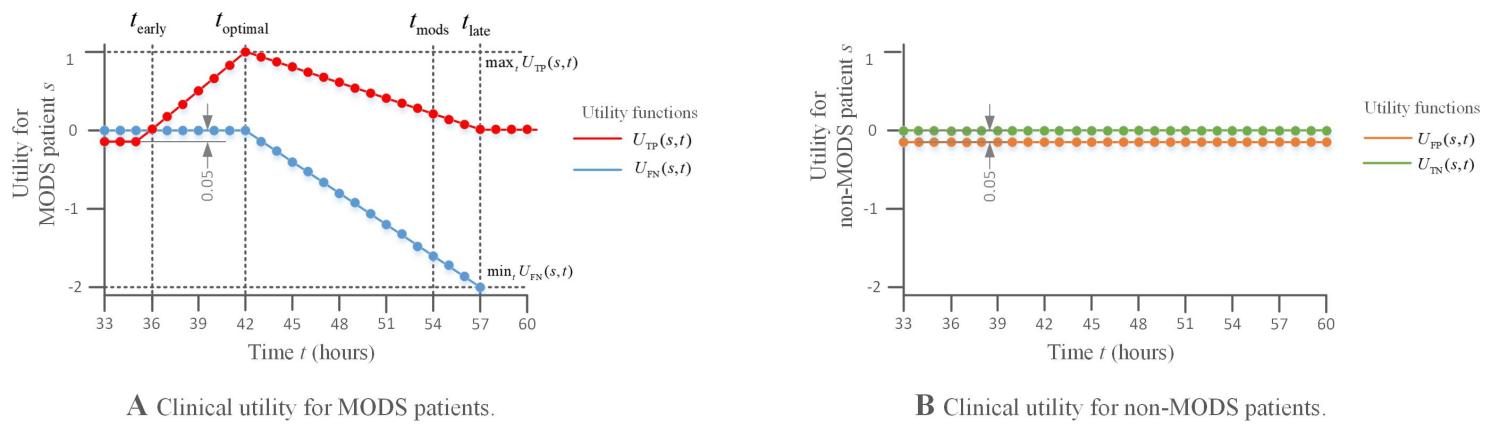


**Figure S1** Diagrams of utility of positive and negative predictions for MODS and non-MODS

patients; the time $t_{MODS}$ = 48 of MODS onset is given as an example.

where $U_{\text{TP}}(s,t)$, $U_{\text{FP}}(s,t)$, $U_{\text{FN}}(s,t)$, and $U_{\text{TN}}(s,t)$ are illustrated in Fig. 1A for an example

patient with MODS and in Fig. 1B for an example patient with non-MODS. According to the multi-segment line in Fig. 1, it is easy to establish functions, and then calculate the score for each point in the Fig. 1. These scores were chosen to reflect the broad clinical realities of MODS detection and treatment, and the actual utility values and time points in (1) and Fig. 1 can be chosen to capture the specific preferences or trade-offs of any particular hospital system.There are the following settings for this study. (i) MODS predictions in patients with MODS that were at least 12 hours before and at most 3 hours after the onset time $t_{MODS}$ of MODS were rewarded with a maximum reward at 12 hours before $t_{MODS}$. Considering that very early predictions may be implausible or unhelpful, MODS predictions that are more than 12 hours before $t_{MODS}$ were slightly penalized.[11]. (ii) Non-MODS predictions in patients with MODS that were later than time $t_{\text{optimal}}$(12 hours before $t_{MODS}$) were critically penalized. (iii) For patients that do not have MODS during their ICU stay, MODS predictions contribute to alarm fatigue and lower confidence in algorithms, antibiotic overuse, and overall poor allocation of hospital attention and resources. Therefore, MODS predictions in non-MODS patients were slightly penalized. (iiii) non-MODS predictions in non-MODS patients were neither rewarded nor penalized. Given an algorithm’s predictions for all hourly time windows T(s) in each patient records, we define total score for an algorithm as the sum

$$U_{total}=\sum_{s\in S} \sum_{t\in T(s)} U(s,t) (2)$$

over all predictions. *S* represents the collection of all patients. For easier interpretability, we normalize (2) so that the optimal algorithm with the highest possible score receives a normalized score of 1 and a completely inactive algorithm that only makes non-MODS predictions receives a normalized score of 0, i.e.,

$$U_{\text{normalized}}=\frac{U_{\text{observed\_utilities }}-U_{\text{inaction\_utilities}}}{U_{\text{best\_utilities}}-U_{\text{inaction\_utilities}}} (3)$$

The $U_{\text{normalized}}$ is utility_score for MODS. $U_{\text{observed\_utilities }}$ is the result calculated according to the observation data. $U_{\text{inaction\_utilities}}$ , $U_{\text{observed\_utilities }}$and $U_{\text{best\_utilities}}$ can be calculated in

https://github.com/physionetchallenges/evaluation-2019/blob/master/evaluate_sepsis_score.py

The reference code gives an early warning 6 hours in advance, while the MODS code gives an early warning 12 hours in advance. The rest are the same.

1. **modified Marshall MODS score**

Table S1: The modified multiple organ dysfunction syndrome (MODS) score

| Organ system | 0 | 1 | 2 | 3 | 4 |
| --- | --- | --- | --- | --- | --- |
| Cardiovascular(heart rate, inotropes, lactate) | ＜=120 | 120-140 | ＞140 | Inotropes | Lactate＞5 |
| Respiratory, Po2/Fio2 | ＞300 | 226-300 | 151-225 | 76-150 | ＜=75 |
| Renal(creatinine, umol/L ) | ＜=100 | 101-200 | 201-350 | 351-500 | ＞500 |
| Central nervous system(Glasgow Coma Score ) | 15 | 13-14 | 10-12 | 7-9 | ＜=6 |
| Hepatic (total bilirubin, umol/L) | ＜=20 | 21-60 | 61-120 | 121-240 | ＞240 |
| Hematologic(platelet count x10^3^) | ＞120 | 81-120 | 51-80 | 21-50 | ＜=20 |

Six organs of the MODS. The cardiovascular component of MODS is defined as follows: 0, heart rate ＜=120 beats per minute (bpm); 1,heart rate 120-140 bpm; 2,heart rate ＞140 bpm; 3, need for inotropes more than dopamine＞3ug/kg/min; 4, serum lactate ＞5mmol/L.

1. **Q-learning**

The pseudocode for the update of Q-table of Q-learning algorithm is as follows.

*Initialize $Q(s,a)$arbitrarily*

*Repeat (for each episode):*

*Initialize* $s$

*Repeat (for each step of episode):*

*Choose$a$from$s$using policy derived from Q (e.g., ε-greedy)*

*Take action $a$, observe $r$,* $s^{'}$

$Q(s,a)\leftarrow Q(s,a)+\alpha\left[r+\gamma\max_{a^{'}}Q(s^{'},a^{'})-Q(s,a)\right] (4)$

$s\leftarrow s^{'}$

*Until $s$ is terminal.*

$s$ indicates current state; $a$, the current action; $s^{'}$, the next state after executing the action $a$;$a^{'}$, action that can be performed in $s^{'}$; $r$, rewards for performing action $a$;$\gamma$, the discount factor; $\alpha$, learning rate. The probability that the agent selects the action$a$ corresponding to the largest value in the Q-table for state $s$is 1-ε; the probability that the agent selects the action$a$ randomly in the Q-table for state $s$is ε.

SuperLearner is a customized two-layer stacked structure. At present, there are few applications of three-layer stacked structure, so we customized a three-layer stacked structure called SubSuperLearner to study whether its prediction performance is higher than that of SuperLearner. of course, SubSuperLearner is one of three-layer stacked structures and the comparison results of these two structures are only valid under the current topology. Q-tables for SuperLearner and SubSuperLearner are as follows.

Table S2: The Q-table for SuperLearner

|  | action 1 | action 2 | action 3 | action 4 | action 5 | action 6 | action 7 | action 8 | action 9 |
| --- | --- | --- | --- | --- | --- | --- | --- | --- | --- |
| states | DWNN | KNN | lightgbm | XGBoost | random forest | AdaBoosting | Naïve Bayes | Decision Tree | Logistic Regression |
| base_1 |  |  |  |  |  |  |  |  |  |
| base_2 |  |  |  |  |  |  |  |  |  |
| base_3 |  |  |  |  |  |  |  |  |  |
| base_4 |  |  |  |  |  |  |  |  |  |
| base_5 |  |  |  |  |  |  |  |  |  |
| base_6 |  |  |  |  |  |  |  |  |  |
| base_7 |  |  |  |  |  |  |  |  |  |
| base_8 |  |  |  |  |  |  |  |  |  |
| meta |  |  |  |  |  |  |  |  |  |

The agent has 9 states, namely base_1~base_8 and meta. In each state, there are 9 actions to choose, which are the nine algorithms corresponding to action 1~action 9. The reward is the AUC value of the candidate SuperLearner on the test.

We use arrows to describe the choices of actions in an episode. “*Initialize $Q(s,a)$arbitrarily*” means that Q-table has been initialized before the first episode, and can be initialized with a value of 0. Each state randomly selects an action (algorithm). When the episode in Table 3 is started, “*Initialize* $s$” means to reset the agent status to base_1. For base_1 line use ε-greedy policy to select actions in the status of this line. Due to base_2~base_8 and the action of meta have been saved in the previous episode or initial random selection or previous step, the AUC value (reward) of this candidate SuperLearner for current step can be calculated. Formula (4) is used to update the value corresponding to the current selected action in Q-table. After this step, the state will automatically change to the next line. Formula (4) not only inclines to the highest reward at present, but also inclines to the best action of the next line (state) at the corresponding maximum value. The update principle corresponding to other states is the same as base_1.

Table S3: The Q-table for SubSuperLearner

|  | action 1 | action 2 | action 3 | action 4 | action 5 | action 6 | action 7 | action 8 | action 9 |
| --- | --- | --- | --- | --- | --- | --- | --- | --- | --- |
| states | DWNN | KNN | lightgbm | XGBoost | random forest | AdaBoosting | Naïve Bayes | Decision Tree | Logistic Regression |
| base1_1 |  |  |  |  |  |  |  |  |  |
| base1_2 |  |  |  |  |  |  |  |  |  |
| base1_3 |  |  |  |  |  |  |  |  |  |
| base1_4 |  |  |  |  |  |  |  |  |  |
| base2_1 |  |  |  |  |  |  |  |  |  |
| base2_2 |  |  |  |  |  |  |  |  |  |
| meta1_1 |  |  |  |  |  |  |  |  |  |
| meta1_2 |  |  |  |  |  |  |  |  |  |
| meta2 |  |  |  |  |  |  |  |  |  |

The Q-table update principle of SubSuperLearner is consistent with that of SuperLearner.

1. **SHAP**

The kernelSHAP can perform group and individual interpretation of various types of algorithms. The SHAP value obtained is based on the results obtained from each individual sample and its corresponding $x_{i}$. For each prediction sample, the model generates a prediction value, the SHAP value is the value assigned to each risk factor in the sample. Suppose the i-th sample is $x_{i}$, the j-th risk factor of the i-th sample is $x_{i,j}$, the model's predicted value for the i-th sample is $y_{i}$ and the baseline of the entire model (usually the mean of the target variable of all subjects) is $y_{base}$. Then the kernelSHAP algorithm obeys the following equation.

$$f(x)=y_{base}+f(x_{i,1})+f(x_{i,2})+...+f(x_{i,k}) (5)$$

is the SHAP value of . is the contribution of individual risk factor j. When , it indicates that this risk factor is a deleterious factor for the instance. When , it means that the risk factor is beneficial for the instance. When ,it means that the risk factor is independent of the instance’s predicted outcome.

For all instances that can form the group, the algorithm takes the mean value of the absolute value of for each instance *i* as the contribution value of group risk factor *j*.

1. **DiCE**

SHAP is used to quantify the contribution of each feature resulting in the current prediction result. DiCE is used to find *counterfactuals*, which meet the requirements of diversity, rationality and can reverse the current prediction result. The key of DiCE modeling is to require the diversity and rationality of *counterfactuals*. In addition, *counterfactuals* generated by DiCE should reverse the original outcome as much as possible. Users get a sufficient number of *counterfactuals*, and the more likely they are to get a more reasonable proposal. But at the same time, we note that the unlimited diversity *counterfactuals* means that several or even dozens of features of the new scheme change at the same time. When users intervene, it is very difficult to control multiple features at the same time, which is obviously unreasonable. Therefore, we need to integrate the diversity and rationality into an objective function to find the best compromise point.

The loss function of diversity is

$$diversity\_loss=-\det(K) (6)$$

Where $K_{i,j}=\frac{1}{1+dist(c_{i},c_{j})}$. $c_{i},(i=1,2,...,k)$ indicates *counterfactuals* with number *i*. *k* represents the number of *counterfactuals* generated. $dist(c_{i},c_{j})$indicates the Manhattan distance from $c_{i}$to $c_{j}$. det (.) represents the determinant function and we add small random perturbations to the diagonal elements for the determinant. When the diversity between counterfacts is more obvious, the value of det (*K*) is greater, so the loss function of diversity contains a negative sign.

The loss function of rationality is

$$rationality\_loss=\frac{1}{k}\sum_{i=1}^{k} dist(c_{i},x) (7)$$

*x* represents the real sample corresponding to the prediction result. $c_{i}$ comes from the change of *x* on one or more features. Obviously, the more similar $c_{i}$ and *x* are, the more reasonable they are, and the smaller their Manhattan distance is.

The loss function of unsuccessful reversal outcome is

$$fail\_loss=\frac{1}{k}\sum_{i=1}^{k} hinge\_yloss(f(c_{i})) (8)$$

$f(c_{i})$ represents the prediction probability of bringing $c_{i}$ into the trained model. We regard $(f(x)-0.5)>0$ as stroke. When $(f(x)-0.5)*(f(c_{i})-0.5)<0$, penalty value namely $hinge\_yloss(f(c_{i}))$ is 0. When $(f(x)-0.5)>0$ and $(f(c_{i})-0.5)>0$, it means there is no reversal of the outcome of stroke, and the penalty value can be $f(c_{i})-0.5$. When $(f(x)-0.5)<0$ and $(f(c_{i})-0.5)<0$, the penalty value can be $0.5-f(c_{i})$. The $hinge\_yloss$ function is not unique. For example, you can increase the penalty value for the case of $(f(x)-0.5)>0$ and $(f(c_{i})-0.5)>0$.

The final loss function is

$$final\_loss=\frac{1}{k}\sum_{i=1}^{k} fail\_loss+\lambda_{1}diversity\_loss+\lambda_{2}rationality\_loss (9)$$

$\lambda_{1}$ and $\lambda_{2}$ are the hyperparameter used to balance the loss function. We use gradient descent method to iterate to reduce $final\_loss$. We initialize all $c_{i}$ randomly, set the number of iterations to 5000 or until $final\_loss$ converges, and finally filter to obtain *counterfactuals* that can reverse the outcome.
